# Clinical significance and burden of carbapenem-resistant Enterobacterales (CRE) colonization acquisition in hospitalized patients

**DOI:** 10.1186/s13756-023-01323-y

**Published:** 2023-11-20

**Authors:** Nasreen Hassoun-Kheir, Khetam Hussien, Marianne Karram, Maram Saffuri, Sally Badaan, Shani Peleg, Worood Aboelhega, Sigal Warman, Tamar Alon, Dina Pollak, Moran Szwarcwort Cohen, Mical Paul

**Affiliations:** 1 Faculty of Medicine Geneva, Geneva University Hospitals, 1205 Geneva, Switzerland; 2 Infectious Diseases and Infection Control Unit, Rambam Health Care Campus, Haifa, Israel; 3 Rappaport Faculty of Medicine, Technion-Israel Institute of Technology, Haifa, Israel; 4 Sackler Faculty of Medicine, School of Public Health, University of Tel-Aviv, Tel-Aviv, Israel; 5 Microbiology Laboratory, Rambam Health Care Campus, Haifa, Israel

**Keywords:** Carbapenem-resistant Enterobacterales, Colonization, Burden, Mortality, Bloodstream infection, Clinically-significant infections

## Abstract

**Background:**

Carbapenem-resistant Enterobacterales (CRE) infections have a significant morbidity and mortality toll. The clinical significance and associated burden of CRE colonization rather than infection state are not frequently investigated. We aimed to assess the outcomes of CRE colonized patients compared to matched controls.

**Methods:**

A secondary analysis of a 1:2 matched case–control study at a tertiary hospital in northern Israel (January-2014 to June-2017). Cases were adults who newly acquired CRE colonization during hospitalization. Controls were inpatients negatively screened for CRE, matched by age, hospitalization division and total days of hospitalization 90 days prior to screening. Our primary outcome was 1-year all-cause mortality. Secondary outcomes included 30-day mortality, diagnosis of any clinical infection, overall days of hospital stay and bloodstream infections all in 1-year follow-up. We estimated crude and propensity score weighted estimates for study outcomes.

**Results:**

We included a total of 1019 patients: 340 CRE colonized and 679 non-colonized controls. After adjustment, CRE colonization was not associated with increased 1-year mortality (weighted OR 0.98, 95% CI 0.64–1.50, p = 0.936). CRE colonized patients had 1.7 times the odds of clinical infection of any cause (weighted odds ratio (OR) 1.65, 95% CI 1.06–2.56, p = 0.025). CRE colonized patients had increased length of hospital stay compared to controls (weighted OR 1.52, 95%CI 1.10–2.10, p < 0.001) among 1-year survivors.

**Conclusions:**

CRE colonization may not be independently associated with mortality but with higher risk of clinical infections and longer hospital stays. Infection prevention and antimicrobial stewardship are of utmost importance to prevent acquisition and infections in colonized patients.

**Supplementary Information:**

The online version contains supplementary material available at 10.1186/s13756-023-01323-y.

## Background

The alarming dissemination of carbapenem resistant Enterobacterales (CRE) mandates better understanding of CRE epidemiology and burden [1]. Infections caused by CRE are associated with 3.4 higher the risk of mortality compared to carbapenem susceptible infections [2]. However, the burden associated with CRE colonization state rather than a clinically-significant infection is rarely investigated. CRE colonization may predispose to adverse outcomes through the risk of a clinically-significant CRE infection for which treatment options are highly limited and through limitations imposed on colonized patients, such as contact isolation, delays in transfer to rehabilitation centers, etc. [3].

In Israel, surveillance for CRE colonization and reporting of newly identified CRE carriers and/or infected patients to the Ministry of Health is mandatory as part of a national policy [4]. Targeted screening of defined high-risk patients for CRE is required on hospital admission, as well as contact tracing. In our center, an expanded surveillance strategy is set in place whereby we perform additional periodic screening in high-risk departments and patient [5].

In this study we aimed to assess whether acquisition of CRE colonization is associated with adverse patient outcomes. We hypothesized that the state of CRE colonization is associated with a higher event rate of all adverse study outcomes through the predisposition to CRE infections and adapted patientcare imposed on colonized patients.

## Methods

### Study setting and design

We conducted a secondary analysis of a 1:2 matched case–control study designed to assess risk factors for CRE acquisition [5]. The study was conducted at Rambam Health Care Campus (RHCC), a 1000-bed primary and tertiary hospital in Haifa, northern Israel, between January 2014 and June 2017.

### Patients

Cases were prospectively identified consecutive patients who acquired CRE colonization and were detected via rectal screening for the first time after being hospitalized for at least 72 h at RHCC. For the current analysis we excluded new CRE carriers diagnosed within 72 h after discharge. Controls were hospitalized patients who were found negative on screening for CRE colonization and never acquired CRE during the study period (screening subsequent to a first negative sample was not required), matched to cases on: age (± 5 years), hospitalization division while screened (intensive care unit, hemato-oncological unit and other hospitalization departments) and total days of hospitalization in RHCC in the preceding 90 days (± 5 days). Index date was defined as the date of rectal swab collection for CRE colonization and negative culture in cases and controls respectively. Index hospitalization was defined as the one in which the patients were screened. The hospital’s CRE surveillance policy, acquisition definitions were previously described [5].

### Microbiological methods

CREs were defined as any Enterobacterales that was found resistant to meropenem (MIC ≥ 4 mcg/ml). CPEs (carbapenemase producing Enterobacterales) were defined with a detectable carbapenemase gene. Isolates were considered non-CP-CRE if no carbapenemase gene was detected and was resistant to meropenem. Rectal swab screening samples were cultured on PD420 CHROMagar KPC plates (HyLabs, Israel). All suspected colonies were tested using Hodge-test and Kirby-Bauer Disk Diffusion Susceptibility Test for meropenem. Polymerase chain reaction (PCR)-based assays were performed only for isolates resistant to meropenem (zone diameter was ≤ 19 mm) and were tested positive for Hodge-test. PCR assays were used to detect carbapenemase genes (blaKPC, blaNDM, blaOXA-48). Enterobacterales that tested positive for Hodge-test and negative for 3 carbapenemase genes, were tested for IMI gene.

### Study variables

Our main exposure was CRE colonization (CPE or non-CP-CRE). As primary outcomes we evaluated all-cause mortality in one year after the index date. Secondary outcomes included 30-day all-cause mortality, and in a 1-year follow-up, diagnosis of any clinical infection during hospitalization, total days of hospital stay in RHCC after index date and clinical infections acquired during hospitalization. Post-discharge mortality data were available through a national registry. Clinical infections were defined according to the CDC'/NHSN criteria [6], and included bloodstream infections (BSIs) and non-bacteremic respiratory and urinary-tract infections. We report on Enterobacterales BSIs (irrespective of antibiotic susceptibility testing results), CRE-BSI and BSI of any pathogen.

### Data collection

RHCC uses a fully electronic patient file (Prometheus, in-house software). We collected retrospectively, manually from patients’ electronic charts, demographic data, co-morbidities assessed at index admission, functional status, and hemoglobin levels measured on index admission. We also assessed antibiotic treatment during the 90 days prior to index date.

### Statistical analysis

We compared CRE colonized patients to controls; categorical variables were presented as absolute numbers and proportions and compared using chi-squared test or Fisher Exact test. Continuous variables were presented as mean ± SD (standard deviation) or median [interquartile range (IQR)] and compared using T-test or non-parametrical test respectively. Missing values for hemoglobin and albumin were imputed using multiple imputations. Survival curves were compared using the Kaplan Meier estimate with a Log rank test.

We estimated unadjusted odds ratios for study outcomes in CRE colonized cases compared to controls using a logistic regression model with a single outcome as the dependent variable and CRE as the exposure. A post-hoc sensitivity analysis using conditional logistic regression was performed to analyze unadjusted effect estimates of primary and secondary outcomes.

We then applied propensity score weighting to estimate adjusted weighted odds ratios for the study outcomes. We modeled a propensity score (PS) using a binary logistic regression model in which the dependent variable was CRE colonization acquisition and independent baseline variables were all variables associated with CRE acquisition, and assessed the model’s overall goodness-of-fit and area under the ROC curve. Overlap weights were calculated as PS for CRE patients and (1-PS) for non-colonized patients [7].

We evaluated difference in cumulative hospital days in RHCC in a 1-year follow-up. We stratified this analysis by 1-year survival status. For this outcome, we used a negative binominal regression model with log link. Finally, we performed a subgroup analysis using similar methods, to evaluate outcomes of CPE carriers and their respective controls. All statistical analyses were performed using SPSS software (ver. 24, SPSS Inc., Chicago IL). The study was approved by the Rambam institutional review board (RMB 0623-16).

## Results

The study included 1019 patients: 340 CRE colonized cases and 679 non-colonized controls. Of the CRE colonized patients 270 (79.4%) carried a carbapenemase producing Enterobacterales (CPE), matched to 540 controls, and 70 carried non-CPE (20.6%), matched to 139 controls. The mean age of all study patients was 63.9 ± 17.4 years with 587/1019 males (57.6%). On the index date, 200/1019 (19.6%) study patients were hospitalized in the ICU, 135/1019 (13.2%) in hemato-oncological departments and 684/1019 (67.1%) patients in other departments. Patient characteristics of the cases and control groups are shown in Table 1. In general, CRE colonized patients had higher comorbidity rates (for all comorbidities, except for malignancy and cerebrovascular disease), longer hospital stays in the 90 days prior to acquisition (despite matching, but within the matching precision range) and higher antibiotic consumption than controls (Table 1).

**Table 1:** Main characteristics of matched cases and controls

Characteristic	CRE colonized patients (n = 340)	Non-colonized patients (n = 679)	P value
Male gender	188 (55.3)	399 (58.8)	0.291
Age (matched) mean ± sd	64 ± 18	64 ± 17	0.400
Hospital division (matched)			> 0.999
ICU	67 (19.7)	133 (19.6)	
Hemato-oncology	45 (13.2)	90 (13.3)	
Other	228 (67.1)	456 (67.2)	
Residence in LTCF	60 (17.6)	83 (12.2)	0.019*
Diabetes mellitus	134 (39.4)	210 (30.9)	0.007*
Malignancy	127 (37.4)	305 (45.1)	0.019*
COPD	29 (8.5)	38 (5.6)	0.078
Chronic kidney disease	30 (8.8)	55 (8.1)	0.704
Liver disease	21 (6.2)	23 (3.4)	0.039*
Cerebrovascular disease	36 (10.6)	85 (12.6)	0.361
Charlson’s comorbidity index median [IQR]	3 [2–6]	3 [2–5]	0.290
Bedridden status	136 (40.0)	213 (31.5)	0.007*
Invasive ventilation	86 (25.3)	135 (19.9)	0.048*
Hemoglobin mean ± sd	9.6 ± 1.8	10.4 ± 2.1	< 0.001*
Total antibiotic days in 90 days prior to index median [IQR]	14 [7–24]	6 [0–18]	< 0.001*
Days of hospital stay in 90 days prior to index (matched) median [IQR] (matched)	23 [12–37]	20 [9–36]	0.012*^a^

*ICU* intensive care unit, *IQR* interquartile range, *LTCF* long term care facility, *COPD* chronic obstructive pulmonary disease, *sd* standard deviation. Percentages are provided if not reported otherwise

*Statistically significant

^a^Within the matching margins

### Propensity score weighting

We included predictors for CRE acquisition in a logistic regression model including demographics, individual comorbidities, physical patient status, colonization with other resistant bacteria, antibiotic treatments and exposure to hospital in the previous 90 days, hemoglobin and albumin levels on index date, study years and exposure to a high CRE colonization pressure as risk factors for CRE acquisition (Additional file 1: Table S1). The model was fit with Hosmer and Lemshow test χ^2^ test of 12.623 and p = 0.126 and area under the ROC of 0.778 (95% CI 0.749–0.804). We assigned weights to the study patients using the model’s probabilities using overlap weighting.

### Primary outcomes

**Fig. 1:**
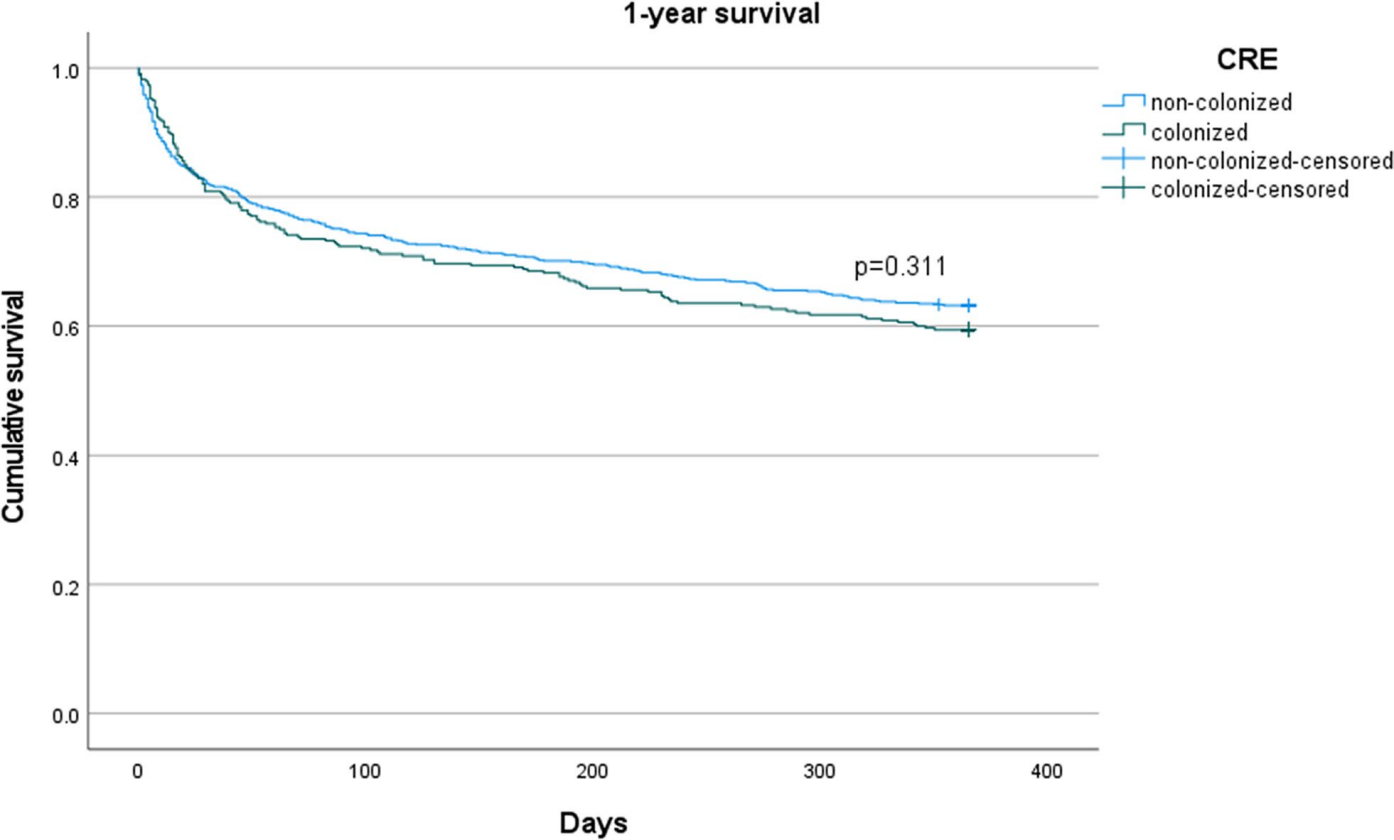
Kaplan Meier curve for 1 year survival

Among CRE carriers, 138 died within one year of the index date (40.6%) compared to 250 (36.8%) controls. Risk factors for 1-year mortality by univariate analysis are shown in Table 2. In the crude analysis, the OR for 1 year mortality was 1.17 (95% CI 0.90–1.53, p = 0.243, Fig. 1). The PS weighted OR for 1-year mortality was 0.98 (95%CI 0.64–1.50, p = 0.936, Table 3).

**Table 2:** Risk factors for 1-year mortality, univariate analysis

Characteristic	Dead in 1 year (n = 388)	Alive in 1 year (n = 631)	P value
Male gender	222 (57.2)	365 (57.8)	0.844
Age mean ± sd	69 ± 15	61 ± 18	< 0.001*
Hospital division			0.350
ICU	85 (21.9)	115 (18.2)	
Hemato-oncology	49 (12.6)	86 (13.6)	
Other	254 (65.5)	430 (68.2)	
Residence in LTCF	71 (18.3)	72 (11.4)	0.003*
Diabetes mellitus	194 (38.4)	195 (30.9)	0.014*
Malignancy	204 (52.6)	228 (36.2)	< 0.001*
COPD	39 (10.1)	28 (4.5)	< 0.001*
Chronic kidney disease	52 (13.4)	33 (5.2)	< 0.001*
Liver disease	20 (5.2)	24 (3.8)	0.303
Cerebrovascular disease	63 (16.2)	58 (9.2)	< 0.001*
Charlson’s comorbidity index median [IQR]	4 [3–7]	3 [1–5]	< 0.001*
Bedridden status	167 (43.0)	182 (28.9)	< 0.001*
Invasive ventilation	108 (27.8)	113 (17.9)	< 0.001*
Hemoglobin (mean ± sd)	9.5 ± 1.8	10.5 ± 2.1	< 0.001*
Total antibiotic days in 90 days prior to index median [IQR]	6 [1–17]	14 [5–30]	< 0.001*

*ICU* intensive care unit, *IQR* interquartile range, *LTCF* long term care facility, *COPD *chronic obstructive pulmonary disease, *sd* standard deviation

*Statistically significant. Percentages are provided if not reported otherwise

**Table 3:** Outcomes of CRE colonized patients compared to controls

Outcome	CRE carriers (n = 340)	Non-colonized patients (n = 679)	Univariate OR (95% CI)	P value	PS weighted OR (95% CI)	P value
1-year mortality	138 (40.6)	250 (36.8)	1.17 (0.90–1.53)	0.243	0.98 (0.64–1.50)	0.936
30-day mortality	65 (19.1)	122 (18.0)	1.08 (0.77–1.51)	0.655	0.82 (0.52–1.53)	0.678
Length of stay in 1 year	18 [14–23]	7 [4–9]				
1-year survivors (n = 631)	18 [5–40]	7 [2–17]	2.05 (1.73–2.44)	< 0.001*	1.52 (1.10–2.10)	0.011*
1-year non- survivors (n = 388)	15 [6–36]	11 [4–31]	1.19 (0.97–1.48)	0.102	0.98 (0.66–1.46)	0.920
Any clinical infection	139 (40.9)	169 (24.6)	2.09 (1.58–2.75)	< 0.001*	1.65 (1.06–2.56)	0.025*
Any BSI	60 (17.6)	56 (8.3)	2.38 (1.61–3.51)	< 0.001*	1.98 (1.07–3.68)	0.030*
Enterobacterales BSI	40 (11.8)	36 (5.3)	2.37 (1.48–3.80)	< 0.001*	2.02 (0.95–4.30)	0.069
CRE BSI**	14 (4.1)	0 (0)	–	–		
CRE UTI**	13 (3.8)	0 (0)				
CRE RTI**	7 (2.1)	0 (0)				

*BSI* bloodstream infection, *UTI* urinary tract infection, *RTI* respiratory tract infection

*Statistically significant,﻿ **A CRE colonized patient might have had a CRE infection in multiple sites during follow-up

### Secondary outcomes

No significant difference in 30-day mortality was observed among CRE colonized patients compared to controls (Table 3). The number of hospital days at RHCC in the year following CRE screening was 2 times higher among survivors in cases compared to controls by crude analysis, with a PS weighted OR of 1.52 (95% CI 1.10–2.10, p = 0.011). CRE colonized patients were diagnosed with clinical infections 1.7 times more often than their controls, (PS weighted OR 1.65, 95% CI 1.06–2.56, p = 0.025). Specifically, the risk of BSI due to any pathogen was significantly higher, PS weighted OR of 1.98, 95% CI 1.07–3.68, p = 0.030). CRE BSIs occurred only among CRE colonized patients, in 14/340 (4.1%). Of note, risk of BSI by any Enterobacterales was also increased among CRE colonized patients compared to controls (PS weighted OR 2.02, 95% CI 0.95–4.30, p = 0.069), however this was not statistically significant. Urinary tract infection and respiratory tract infection due to CRE occurred in 13/340 patients (3.8%) and 7/340 patients (2.1%) respectively (Table 3).

A sensitivity analysis using conditional logistic regression accounting for matching, resulted in similarly increased effect estimates for both primary and secondary outcomes (Additional file 1: Table S2). In a subgroup analysis, including CPE carriers and their respective controls (n = 810 patients), increased risk for clinical infections, any BSIs and Enterobacterales BSIs was also observed in unadjusted analysis as well as longer length of hospital stay for 1-year survivors (Additional file 1: Table S3). In PS-weighted analyses, strong evidence to support this increased risk remained only for any BSIs and clinical infections with PS-weighted OR of 2.36 (95% CI 1.11–5.00, p = 0.025) and 1.71 (95% CI 1.03–2.85, p = 0.038) respectively.

## Discussion

Colonization with CRE was not associated with higher 1-year mortality in our study. Similar results were observed for shorter follow-up of 30-day mortality. Nonetheless, CRE colonized patients experienced higher frequency of infections of any cause with a PS-weighted OR of 1.65 (95% CI 1.06–2.56). Of interest is the increased risk for BSI, that was twice the risk in CRE carriers compared to their respective controls. Days of hospital stay in the year following CRE acquisition was higher among CRE colonized patients compared to controls in those who survived, PS-weighted OR 1.52 (95% CI 1. 01–2.10). In a subgroup analysis of CPE carriers and their controls, significantly increased risks for BSIs with PS-weighted OR of 2.36 (95% CI1.11–5.00) and for clinical infections with PS weighted OR of 1.71 (95% CI 1.03–2.85, p = 0.038) were observed.

CRE colonization can persist for a long duration. In a meta-analysis assessing CRE colonization duration, 73.9% (95% CI 64–81.8) and 55.2% (95% CI 37.7–71.9) of patients continued carriage at 1 and 6 months after colonization detection [8]. Long-term mortality is the ultimate toll of CRE carriage, encompassing the burden of CRE infections that cannot be treated optimally, isolation in healthcare facilities and delays or even avoidance of certain procedures and opportunities due to the carriage state and contact isolation.

Similar to our findings, colonization with CRE was no longer significantly associated with increased mortality after adjusting for patients’ characteristics in previous studies. CRE colonization was shown to be a strong predictor of CRE infection in intensive care unit (ICU) patients but was not associated with 90 days all-cause mortality [9]. A higher OR for mortality was observed (adjusted OR 2.3, 95% CI 0.1–5.3) in that study, evaluating ICU patients, but only 36 CRE carriers were included [10]. In another study comparing 164 CRE carriers to 62 controls in the ICU setting, CRE colonization was not associated with mortality but with increased length of hospital stay [10]. Excess mortality in CRE colonized patents was observed in specific patient groups, following liver transplantation [11], and patients with acute leukemia [11, 12]. These patients might cope worse with clinical CRE infection, relying more heavily on effective antibiotic therapy that was unavailable for CRE. In contrast to previous studies, in our study, we analyzed the association between CRE colonization and mortality in a large and heterogenous patient group.

As to the risk of developing clinical infection, most studies focus on the risk of CRE-infection among colonized patients; a systematic review by Tischendrof et al. described a cumulative incidence of 16.5% for CRE infection among colonized patients [13]. Moreover, numerous studies assessing risk for CRE-infection include patients diagnosed both in the colonization and the infection states. We describe for the first time the risk of clinical infection, irrespective of the causative pathogen measured following acquisition of CRE colonization. Our current findings show a strong association between CRE acquisition and development of clinical infections. This might be related to the limitations imposed on CRE carriers due to isolation requirement, or a reflection of the differences between CRE carriers and non-carriers. We attempted to overcome the inherent differences between CRE colonized and non-colonized patients by selecting similar controls who were screened for CRE, and by adjusting for confounders using propensity score overlap weighting [7]. When CRE causes a clinical infection, as in 4% of CRE carriers in our study who developed CRE BSI, the burden of disease becomes even more evident due to the limited treatment options available. Broad-spectrum antibiotics are often prescribed, promoting further selective pressure both in the same patient and in other patients in the corresponding hospitalization ward.

In our study, CRE colonized patients who survived for 1 year, required longer hospital stays than controls. We measured days of hospital stay starting from colonization detection (or index day for controls) and adjusted it for previous hospitalization days through matching, as well as other confounders. The increased hospital stay of CRE colonized patients has multiple implications: first, the potential of cross-transmission and spreading CRE to other hospitalized patients is increased. This is of special concern in light of recent evidence on horizontal plasmid transfer occurring almost in every colonized patient [14], and might be leading to underestimation of cross-transmission risks in the current literature. Implementation of antimicrobial stewardship and infection prevention interventions could not be overemphasized in CRE carrier patients. Moreover, the resources and dedicated staff needed for treating these patients are higher, and lastly the daily isolation costs can sum up to significant amounts [15]. Of note, in CPE carriers the longer hospital stays among 1-year survivors was not maintained, hinting to differences between CPE and non-CP-CRE carriers.

The main strengths of our investigation build up upon the fact that intensive screening enabled detection of CRE colonization state prior to infection in a large number of patients, in an endemic setting. We included a large and heterogenous sample of patients acquiring colonization in different settings (ICU, hemotological and other wards). We evaluated important outcomes relevant in both patients’ and hospital’s perspectives. We used PS overlap weighting to overcome the inherent differences between CRE colonized patients and controls; and were able to define excess infection risk and excess length of stay associated with CRE colonization, adding up to the already known burden of CRE infection.

Despite our study findings, caution should be taken when linking severe outcomes solely to the state of CRE colonization, as causality cannot be determined. It is not clear to what extent the colonizing bacterium plays a role in the context of host factors. Patients who acquire CRE, and multidrug resistant organisms in general, tend to have high morbidity rates prolonged exposure to the healthcare system, often carry invasive instrumentation and are prone to adverse health outcomes. Yet, this does not need to influence the need for infection prevention and antimicrobial stewardship in these patients. As a single center study, our findings might not be generalizable to other settings. Despite our efforts, the generated estimates might still be subject to residual confounding. We did not follow the patients outside our hospital, however, as it is the only tertiary hospital in northern Israel, patients tend to be re-admitted to the same facility, but we still might have missed community-onset infections that did not require hospitalization. Last, the majority of the included cases carried CPE rather than non-CP-CRE, thus our findings tend to less reflect the latter.

No decolonization approach had achieved long term effect on CRE carriage in previous studies [8]. Further research should tackle novel decolonization strategies. Vaccines could be an interesting option for colonized or at-risk patients, but the current pipeline is lacking candidate agents for Enterobacterales in advanced stages of development [16]. Further research should evaluate optimal interventions to limit cross-transmissions in the healthcare settings. The optimal strategies to screen patients for multi-drug resistant organisms and CRE colonization, in particular, should be defined in cluster randomized trials. Future studies should also define better indicators for increased CRE cross-transmission rather than occurrence of CRE clinical infections and cumbersome and costly patient screening, such as wastewater and environmental surveillance. Finally, the actual costs of CRE colonization and cost-effectiveness of surveillance and infection prevention efforts should be investigated.

In summary, in our study CRE colonization was not associated with mortality but with higher risk of clinical infections and longer hospital stays. Infection prevention and antimicrobial stewardship are of utmost importance to prevent colonization and prevent infections in colonized patients.

### Supplementary Information

**Additional file 1**. Supplementary materials.

## Data Availability

Not applicable.

## Abbreviations

BSI: Bloodstream infection
CI: Confidence interval
COPD: Chronic obstructive pulmonary disease.
CPE: Carbapenemase producing Enterobacterales
CRE: Carbapenem-resistant Enterobacterales
ICU: Intensive care unit
IQR: Interquartile range
LTCF: Long term care facility
MDRO: Multidrug resistant organism
Non-CP-CRE: Non carbapenemase producing (CPE or non-CP-CRE)
OR: Odds ratio
PS: Propensity score
RHCC: Rambam Health Care Campus
RTI: Respiratory tract infection
UTI: Urinary tract infection
