# Supplementary material for: Clinical significance and burden of carbapenem-resistant Enterobacterales (CRE) colonization acquisition in hospitalized patients
Source: Antimicrob Resist Infect Control. 2023 Nov 20;12:129. doi: 10.1186/s13756-023-01323-y (PMC10658805; doi:10.1186/s13756-023-01323-y)
Supplement: Supplementary file 1 — Additional file 1. Supplementary materials. [file 13756_2023_1323_MOESM1_ESM.docx]

Table S1 propensity score model for the prediction of CRE acquisition among hospitalized patients

| Risk factors | OR | lower 95% CI | upper 95%CI | p value |
| --- | --- | --- | --- | --- |
| Age (years) | 1.00 | 0.99 | 1.01 | 0.547 |
| Female | 1.46 | 1.06 | 2.00 | **0.019** |
| *hospital division*  ICU (reference) |  |  |  | **<0.001** |
| Hematology | 0.64 | 0.34 | 1.18 | 0.151 |
| Other hospital departments | 1.93 | 1.20 | 3.11 | **0.007** |
| Number of negative swabs during 90 days prior to screening | 1.18 | 1.10 | 1.28 | **<0.001** |
| Chronic dialysis | 0.62 | 0.22 | 1.75 | 0.371 |
| Bedridden status at baseline | 0.99 | 0.68 | 1.45 | 0.966 |
| Pressure ulcer | 0.75 | 0.49 | 1.16 | 0.196 |
| Total days in hospital 90 days prior to screening | 0.96 | 0.95 | 0.97 | **<0.001** |
| Diabetes Miletus | 1.44 | 1.03 | 2.03 | **0.035** |
| *CRE exposure in 90 days prior to screening*  No CRE exposure (reference) |  |  |  | **0.029** |
| Low CRE exposure | 1.02 | 0.70 | 1.50 | 0.912 |
| High CRE exposure | 1.63 | 1.10 | 2.41 | **0.015** |
| Prior MDRO colonization | 1.12 | 0.67 | 1.88 | 0.659 |
| Carbapenem treatment days | 1.02 | 1.00 | 1.05 | 0.057 |
| Specialist consults in index hospitalization prior to screening  No consults (reference) |  |  |  | **<0.001** |
| 1-3 consults | 1.97 | 1.33 | 2.92 | **0.001** |
| More than 3 consults | 2.66 | 1.76 | 4.01 | **<0.001** |
| Referral from long term care facility | 1.51 | 0.96 | 2.38 | 0.074 |
| Cephalosporin treatments days | 1.04 | 1.02 | 1.06 | **<0.001** |
| Hemoglobin (gr/dl) | 0.90 | 0.82 | 0.99 | **0.030** |
| Arab ethnicity | 2.39 | 1.63 | 3.51 | **<0.001** |
| Liver disease | 1.62 | 0.92 | 2.84 | 0.095 |
| Congestive heart failure | 0.81 | 0.54 | 1.23 | 0.330 |
| Ischemic heart disease | 1.27 | 0.83 | 1.94 | 0.270 |
| Chronic obstructive pulmonary disease | 1.35 | 0.74 | 2.47 | 0.333 |
| Peripheral vascular disease | 0.84 | 0.47 | 1.50 | 0.560 |
| Cerebrovascular disease | 0.79 | 0.49 | 1.30 | 0.359 |
| Dementia | 0.79 | 0.41 | 1.55 | 0.496 |
| Solid Tumor | 0.53 | 0.39 | 0.73 | **<0.001** |
| chronic proton pump inhibitors treatment | 1.27 | 0.92 | 1.74 | 0.141 |
| Albumin (gr/dl) | 0.67 | 0.52 | 0.86 | **0.002** |
| Study years (2014-15 vs. 2016-17) | 0.74 | 0.54 | 1.02 | 0.063 |

Table S2 – Sensitivity analysis using conditional logistic regression for unadjusted effect estimates for CRE colonization on study outcomes – accounting for matching.

| Outcome | CRE carriers (n=340) | Non-colonized patients (n=679) | Unadjusted OR (95% CI) | P value |
| --- | --- | --- | --- | --- |
| 1-year mortality | 138 (40.6) | 250 (36.8) | 1.1 (0.89-1.35) | 0.370 |
| 30-day mortality | 65 (19.1) | 122 (18) | 1.06 (0.79-1.43) | 0.698 |
| Any clinical infection | 139 (40.9) | 169 (24.6) | 1.65 (1.31-2.06) | **<0.001** |
| Any BSI | 60 (17.6) | 56 (8.3) | 2.14 (1.49-3.08) | **<0.001** |
| Enterobacterales BSI | 40 (11.8) | 36 (5.3) | 2.22 (1.42-3.49) | **<0.001** |

Table S3 – Subgroup analysis, outcomes of CPE colonized patients compared to controls.

| Outcome | CPE carriers (n=270) | Non-colonized patients (n=540) | Univariate OR (95% CI) | P value | PS weighted OR (95% CI) | P value |
| --- | --- | --- | --- | --- | --- | --- |
| 1-year mortality | 107 (39.6) | 189 (35.0) | 1.22 (0.90-1.65) | 0.197 | 0.95 (0.59-1.55) | 0.848 |
| 30-day mortality | 50 (18.5) | 95 (17.5) | 1.07 (0.73-1.56) | 0.746 | 0.87 (0.47-1.62) | 0.656 |
| Length of stay in 1 year | 15 [5-35] | 7 [2-19] |  |  |  |  |
| 1-year survivors (n=514) | 17 [4-34] | 6 [2-15] | 1.94 (1.61-2.35) | **<0.001** | 1.23 (0.85-1.80) | 0.278 |
| 1-year non-survivors (n=296) | 13 [5-35] | 10 [3-30] | 1.09 (0.86-1.39) | **0.469** | 0.84 (0.51-1.37) | 0.479 |
| Any clinical infection | 104 (38.5) | 123 (22.8) | 2.12 (1.55-2.92) | **<0.001** | 1.71 (1.03-2.85) | **0.038** |
| Any BSI | 44 (16.3) | 35 (6.5) | 2.80 (1.75-4.48) | **<0.001** | 2.36 (1.11-5.00) | **0.025** |
| Enterobacterales BSI | 30 (11.1) | 23 (4.3) | 2.80 (1.59-4.92) | **<0.001** | 2.08 (0.86-5.03) | 0.106 |
| CRE BSI | 13 (4.8) | - | - | - |  |  |
| CRE UTI | 12 (4.4) | - | - | - |  |  |
| CRE RTI | 6 (2.2) |  |  |  |  |  |
